# Intracardiac thrombi in pediatrics: anticoagulation approach and treatment outcomes

**DOI:** 10.1016/j.rpth.2023.102266

**Published:** 2023-11-24

**Authors:** Shreya Agarwal, Eman Abdelghani, Joseph R. Stanek, Amanda Sankar, Clifford L. Cua, Bryce A. Kerlin, Vilmarie Rodriguez

**Affiliations:** 1 Division of Hematology, Department of Pediatrics, UCSF Benioff Children’s Hospital, San Francisco, California, USA; 2 Pediatric Hematology, Indiana Hemophilia Treatment Center, Indianapolis, Indiana, USA; 3 Division of Hematology/Oncology/BMT, Department of Pediatrics, The Ohio State University College of Medicine, Columbus, Ohio, USA; 4 Biostatistics Resource at Nationwide Children’s Hospital, Columbus, Ohio, USA; 5 Division of Cardiology, Department of Pediatrics, The Ohio State University, Columbus, Ohio, USA; 6 Center for Clinical and Translational Research, Abigail Wexner Research Institute, Nationwide Children’s Hospital, Columbus, Ohio, USA

**Keywords:** anticoagulation, congenital heart disease, intracardiac thrombi, pediatrics, thrombosis

## Abstract

**Background:**

Intracardiac thrombi (ICT) are associated with significant morbidity and mortality. Anticoagulation is the first line of treatment and may be complemented by thrombectomy or thrombolysis. However, optimal anticoagulant duration remains ill-defined. High-risk features of ICT that may warrant long-term anticoagulation therapy have not been established.

**Objectives:**

To describe anticoagulation duration and patterns of ICT resolution. To identify potential risk factors for persistent ICT despite anticoagulation.

**Methods:**

A single-institution retrospective chart review identified patients diagnosed with ICT by echocardiogram between January 2014 and March 2022. Descriptive statistics and logistic regression were used.

**Results:**

Fifty-one patients with ICT were identified. Median age at diagnosis was 9.2 years (IQR, 0.4-15.2). The most common underlying diagnoses were congenital heart disease (41%), infection (25%), and malignancy (24%). The majority of ICT were in the right atrium (*n* = 30). The median longest ICT dimension was 1.5 cm (range, 0.4-4.0). The median duration of anticoagulation was 4.3 months (IQR, 2.2-9.1). Among 48 patients who received anticoagulation as first-line treatment, 32 had partial or complete response with 3 to 6 months of anticoagulation, while remaining 16 patients had no response to anticoagulation. Patients with a central venous line had a delayed resolution of ICT [hazards ratio = 0.45 (95% CI, 0.22-0.93)].

**Conclusion:**

Our study demonstrates the wide variability in duration of anticoagulation for children with ICT. Majority of the individuals benefit from 3-to-6 month treatment; however, individuals with a central venous line may benefit from a longer course of anticoagulation. Further large-scale studies are recommended to validate our findings.

## Essentials

• Optimal duration of anticoagulation for intracardiac thrombi is not well established.
• We conducted a single-center retrospective study to observe the response to anticoagulation.
• There was wide variability in the duration of anticoagulation; median duration was 4 months.
• Patients with a central venous line had a delayed response to anticoagulation.

## 1 Introduction

Intracardiac thrombi (ICT) are rare in children but carry a significant risk of morbidity and mortality [1]. In the pediatric population, the majority of ICT occur in the setting of a provoking risk factor such as a central venous line (CVL), low cardiac output, or a prothrombotic comorbidity [2,3]. Anticoagulation is the mainstay of treatment which may be supplemented by thrombectomy or thrombolysis on a case-by-case basis [4,5]. However, determining the optimal duration of anticoagulation remains challenging. The American College of Chest Physicians (CHEST) 2012 guidelines recommend a minimum of 3 months of anticoagulation for right atrial thrombus [6], but there are no recommendations regarding when to consider longer duration of therapy. Similarly, the 2018 *American Society of Hematology* pediatric guidelines for venous thromboembolism (VTE) management recommend anticoagulation for right atrial thrombosis, acknowledging the lack of pediatric evidence regarding the duration of treatment and the impact of the thrombus size and mobility [7]. This makes it difficult for providers to make an informed decision regarding the appropriate length of anticoagulation, therefore, results in variability in the management of these patients.

The objective of this single-center study was to describe the duration of anticoagulation therapy and patterns of resolution for patients with ICT to begin to fill the knowledge gap regarding optimal clinical management in pediatric ICT. Furthermore, we aimed to identify potential risk factors for lack of resolution of the ICT with anticoagulation and describe thromboembolic and bleeding complications in this population.

## 2 Methods

### 2.1 Study cohort

This study was approved by the Nationwide Children’s Hospital Institutional Review Board.

We conducted a retrospective single-center cohort study of children aged <22 years at the time of ICT diagnosis between January 1, 2014, and March 15, 2022, identified on echocardiogram.

Potential subjects were identified by reviewing the Nationwide Children’s Hospital heart center database and using the keyword “thrombus” in the coded diagnosis, the summary, or the indication for the echocardiogram. Electronic medical records of each potential subject were then reviewed manually to confirm the presence of an ICT and extract clinical and demographic data. Patients were excluded if there was inconclusive ICT on echocardiogram or if the location of thrombus was extracardiac. Patients with left ventricular assist device and extracorporeal membrane oxygenation were also excluded due to the difference in flow dynamics in those patients.

### 2.2 Data collection

Data were collected regarding patient demographics; clinical characteristics of the ICT including size, mobility, location, and left ventricular ejection fraction (LVEF); prothrombotic risk factors such as presence of CVL, underlying congenital heart disease (CHD), recent surgery, malignancy, and active infection. Patient charts were also reviewed for treatment approach including first line of management (anticoagulation, thrombectomy, or thrombolysis), first-line anticoagulant, and duration of anticoagulation. Primary outcomes of interest were resolution of the ICT on echocardiogram and clinical variables associated with poor response to anticoagulation. Secondary outcomes included thromboembolic events like stroke, pulmonary embolism, and bleeding events while on anticoagulation.

### 2.3 Study definitions

The date of ICT was defined as the date when ICT was first identified on the echocardiogram. Complete resolution (CR) was defined as no evidence of the ICT on repeat echocardiogram while partial resolution (PR) was defined as any decrease in the size of the ICT. Major bleeding and clinically relevant non major bleeding were defined as per the *International Society of Thrombosis and Hemostasis* criteria [8]. For patients receiving indefinite anticoagulation, duration of anticoagulation was censored at the end of the data review (October 2022). Low LVEF was defined as a LVEF of <50% [9,10].

### 2.4 Statistical analysis

Descriptive statistics were used to assess the baseline demographics and clinical characteristics with median and IQR for continuous variables and frequencies and percentage for categorical variables. Total duration of anticoagulation was calculated as median and IQR. Subjects were censored at the end of study period, or the date of their last contact in the hospital Electronic medical records. Swimmer plot was used to graphically represent each patient’s timing of response to anticoagulation and amount of follow-up. On the subset of patients who were treated with anticoagulation, associations between clinical variables and the time to ICT resolution were studied with a competing risk model with time to CR or PR as the event of interest and death as a competing event. Patients whose ICT did not resolve and were surviving were considered censored during their last echocardiogram. These analyses were presented as hazard ratios and corresponding 95% CIs. Owing to the limited sample size, multivariable regression models were not considered appropriate. *P* value of <.05 was considered statistically significant. Analyses were completed using SAS software, version 9.4 (SAS Institute).

## 3 Results

### 3.1 Study population and ICT characteristics

**Figure 1:**
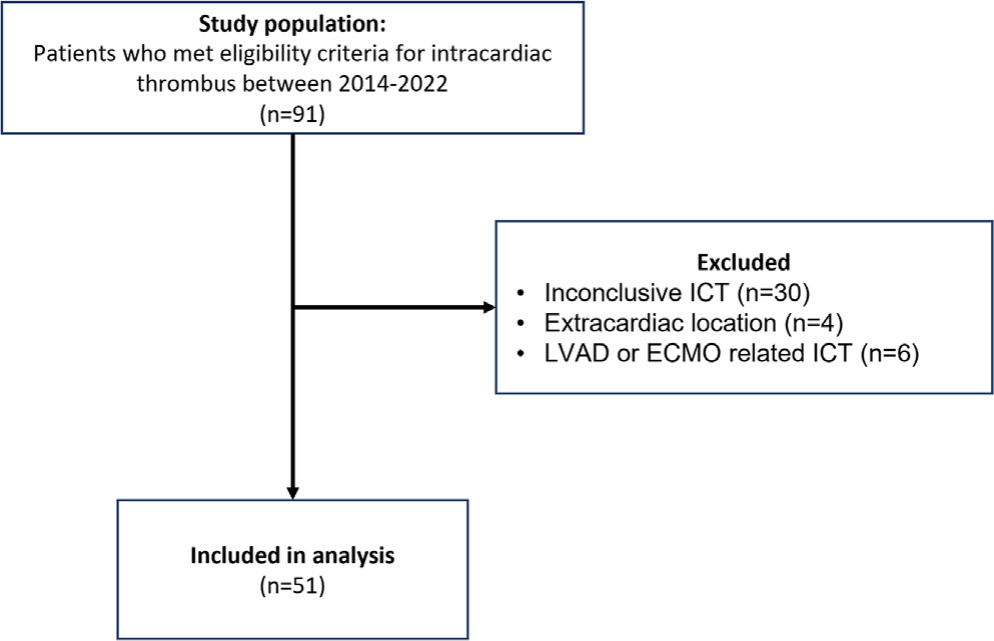
Consort diagram of children evaluated for inclusion in the study between January 2014 and March 2022.

Between January 2014 and March 2022, a total of 91 eligible patients were initially identified and 51 patients were ultimately included in the final analysis (Figure 1). Table 1 summarizes the demographic and clinical characteristics of the cohort. Majority were male (*n* = 35, 67%) and the median age at diagnosis was 9.2 years (IQR, 0.4-15.2 years) (Table 1). Only 4 patients (7.8%) were symptomatic from the ICT, while remainder 47 (92.2%) were identified on routine echocardiogram. Common underlying risk factors for the ICT included presence of CVL (73%), CHD (41%), malignancy (25%), and active infection (24%). Of the 21 patients with CHD, 5 (24%) had dilated cardiomyopathy and 1 had hypertrophic cardiomyopathy (5%). Low LVEF was observed in one third of the cohort (*n* = 17, 33%). Twenty-two percent of the patients had a concurrent contiguous or non-contiguous deep vein thrombosis at the time of the ICT diagnosis.

Abbreviations used: LVAD-Left ventricular assist device, ECMO-Extracorporeal membrane oxygenation.

**Table 1:** Demographics and underlying clinical characteristics.

Characteristic	*N* = 51 *n* (%)
Male sex	35 (68.6)
Age at diagnosis, y	9.2 (0.4-15.2)
Race	
Non-Hispanic White	31 (60.8)
Non-Hispanic Black/African American	15 (29.4)
Asian	3 (5.9)
American Indian	1 (2.0)
Hispanic	1 (2.0)
Underlying primary diagnosis	
Congenital heart disease	21 (41.1)
Cancer	13 (25.5)
Infection	12 (23.6)
Other	5 (9.8)
Presence of central venous line	37 (72.5)
Concurrent deep vein thrombosis^a^	11 (21.6)

LE, lower extremity; UE, upper extremity.

a Deep vein thrombosis locations include 1 bilateral LE, 3 left LE, 4 right LE, 3 right UE, 1 bilateral LE + right UE.

The most common location of the ICT was in the right atrium (*n* = 30, 59%), followed by left ventricle (*n* = 9, 18%), right ventricle (*n* = 6, 12%), and left atrium (*n* = 4, 8%). In the remaining 2 patients, ICT were observed in mitral valve or as biventricular thrombus. Of the 30 patients with right atrium ICT, 24 patients had a CVL at the time. Median longest dimension of the ICT was 1.5 cm (IQR, 1.0-2.1 cm). Around half of the ICT were noted to be mobile on the echocardiogram (*n* = 28, 55%). Table 2 summarizes the ICT features. Median time from infection to ICT diagnosis was 7 days (IQR, 2-15 days), CVL placement to ICT diagnosis was 14 days (IQR, 3-99 days) while cardiac surgery to ICT diagnosis was 21 days (IQR, 8-1192 days).

**Table 2:** Intracardiac thrombus information.

Intracardiac thrombus information	*N* = 51 *n* (%)
Location	
Right atrium	30 (58.8)
Right ventricle	6 (11.8)
Left atrium	4 (7.8)
Left ventricle	9 (17.6)
Others^a^	2 (3.9)
Side	
Right	37 (74.0)
Left	13 (26.0)
Mobile intracardiac thrombus	28 (54.9)
Evidence of patent foramen ovale	7 (13.7)
Decreased left ventricle ejection fraction	17 (33.3)
Longest dimension (in cm)	1.5 (1.0-2.1)
Anticoagulation duration (mo)	4.3 (2.2-9.1)

a Others includes 1 mitral valve, 1 biventricular thrombus.

### 3.2 Anticoagulation approach and response to treatment

None of these patients were on anticoagulation prior to the diagnosis of ICT. Almost all patients received anticoagulation as the first line of treatment for ICT (48/51, 94%). The other 3 patients underwent mechanical thrombectomy as first-line treatment. Five patients received thrombolysis in addition to anticoagulation. The most common first-line anticoagulant agents used were enoxaparin (*n* = 24, 50%) and unfractionated heparin (*n* = 18, 38%). Other agents used include bivalirudin (*n* = 3, 6%), warfarin (*n* = 1, 2%), rivaroxaban (*n* = 1, 2%), and apixaban (*n* = 1, 2%). Overall duration of anticoagulation was variable with a median duration of anticoagulation was 4.3 months (IQR, 2.2-9.1 months). Nine patients were receiving indefinite anticoagulation at the time of analysis, with either warfarin (*n* = 7, 78%), enoxaparin (*n* = 1, 11%) or apixaban (*n* = 1, 11%). Median follow-up period was 374 days (IQR, 93-637 days).

**Figure 2:**
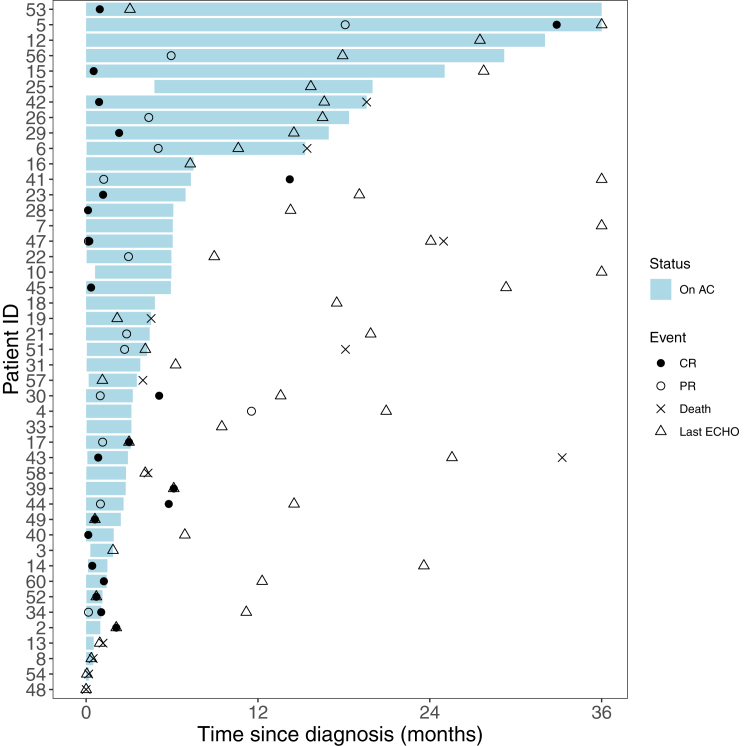
Swimmer plot describing each patient’s duration of anticoagulation and response to treatment.

**Figure 3:**
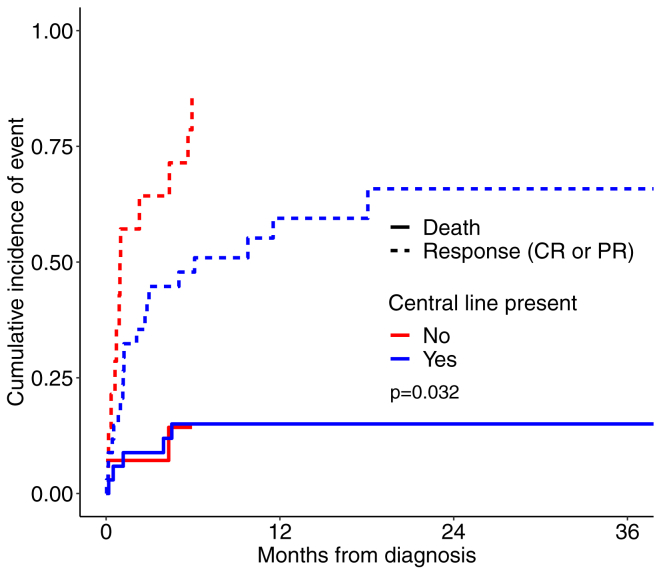
Cumulative incidence plot of response in those with and without a central venous line with death as a competing event.

Among the 48 patients who received at least some anticoagulation, a total of 32 patients achieved CR or PR, while 16 had no response to anticoagulation at the end of their anticoagulation course. Figure 2 demonstrates each patient’s duration of anticoagulation and time to response and highlights the variability in treatment duration. Among the 16 patients with no response to anticoagulation, median duration of anticoagulation was 3.6 months (IQR, 1.1-5.7). Seven of these patients died. Among the 9 surviving patients, median follow-up period was 15.7 months (IQR, 7.3-27.5 months). Among the 32 patients with a partial or complete response, there were 5 deaths, and the median duration of anticoagulation and follow-up were 5.2 months (IQR, 2.5-16.1) and 14.4 months (IQR, 6.5-22.3), respectively. In those with an observed response, 25 patients (78%) were found to have some response within 3 months of initiating anticoagulation, while 3 patients had a response 3-to-6 months after starting anticoagulation (10%). One patient required >1 year of anticoagulation before any response was observed. This patient had CHD (hypoplastic left heart syndrome s/p Fontan procedure) and was found to have partial response after almost 18 months of anticoagulation and complete response by 27 months. Three patients had responses off anticoagulation. These 3 patients had received 1, 2.8, and 3.2 months of anticoagulation, respectively. ICT resolution occurred 34, 102, and 255 days after stopping anticoagulation. Table 3 compares the factors associated with time to ICT resolution in patients who received anticoagulation while accounting for death as a competing event. The only significant finding was that presence of CVL was associated with a delayed time to ICT resolution (hazard ratio = 0.45 [95% CI, 0.22-0.93]) (Table 3, Figure 3).

**Table 3:** Factors associated with ICT resolution in patients who received anticoagulation.

Clinical characteristic	Resolved (CR/PR) *N* = 32 *n* (%)	No resolution *N* = 16 *n* (%)	Hazards ratio (95% CI)
Age (y)	8.9 (1.4-15.5)	13.7 (0.4-14.7)	1.0 (0.95-1.04)
ICT size (cm)	1.6 (1.0-2.3)	1.4 (1.1-2.0)	0.92 (0.58-1.47)
ICT right side (vs left)^a^	24 (77.4)	11 (68.8)	1.31 (0.55-3.10)
Mobile ICT	18 (56.3)	9 (56.3)	0.94 (0.47-1.88)
Normal LVEF^b^	8 (25.0)	9 (56.3)	1.71 (0.69-4.21)
Congenital heart disease	13 (40.6)	6 (37.5)	1.18 (0.58-2.41)
Cancer	8 (25.0)	4 (25.0)	1.12 (0.51-2.46)
Deep vein thrombosis	8 (25.0)	3 (18.8)	1.14 (0.55-2.37)
Infection	7 (21.9)	4 (25.0)	0.88 (0.39-1.98)
Central venous line	20 (62.5)	14 (87.5)	0.45 (0.22-0.93)
Duration of anticoagulation (months)	5.2 (2.5-16.1)	3.6 (1.1-5.7)	
Median duration of follow-up (mo) (time from diagnosis to last echocardiogram)	14.4 (6.5-22.3)	5.2 (1.1-16.6)	

CR, complete response; ICT, intracardiac thrombus; LVEF, left ventricle ejection fraction; PR, partial response.

a Patient with bilateral ICT excluded from this analysis.

b Decreased LVEF defined as LVEF <50%. Hazard ratios calculated by modeling time to earliest response (CR or PR) with death as a competing event. Patients without a response or death were considered censored during their last echocardiogram.

### 3.3 Thrombectomy for ICT

There were 3 patients who underwent thrombectomy. Patient 1 had left atrium ICT with the longest dimension of 2.1 cm. This patient also developed cardioembolic stroke the same day. The course was further complicated by thrombocytopenia with platelet counts of <50,000. Due to concern for hemorrhagic conversion with anticoagulation and in the setting of thrombocytopenia, plan was made to proceed with mechanical thrombectomy only. Patient 2 had ICT at the mitral valve with the longest dimension of 0.5 cm. This patient had mitral and pulmonary valve stenosis and underwent thrombectomy with mitral valve replacement on the day of ICT diagnosis. Anticoagulation with warfarin was started after thrombectomy. Patient 3 had ICT at the right atrium, with longest dimension of 2.7 cm. There was concern for myxoma versus ICT; therefore, the patient underwent resection, and pathological findings confirmed thrombus. All the 3 patients had CR after thrombectomy.

### 3.4 Thromboembolic and bleeding complications and mortality

There were 5 (9.8%) stroke events observed. Of the 5 patients diagnosed with a stroke, 4 had a left sided ICT (3 left ventricles and 1 left atrium) and one was in the right atrium. Three of the 5 ICTs were mobile. Three patients had a stroke the same day as ICT diagnosis and were not receiving anticoagulation at the time, while the remainder 2 patients were receiving anticoagulation at the time of stroke. Due to small number of events, further risk factor analysis was not performed. The overall mortality rate was 23.5% (*n* = 12) with 4 deaths within 1 month of ICT diagnosis, and the remainder 8 deaths occurred ≥6 months after diagnosis. However, none of these deaths were deemed to be related to ICT and/or anticoagulation. The rate of major and clinically relevant non major bleeding events were 8% (*n* = 4). These bleeding events include hemoperitoneum (*n* = 1), hemorrhagic stroke (*n* = 1), intracranial hemorrhage (*n* = 1) and pericardial effusion with tamponade requiring pericardiocentesis and pericardial drain (*n* = 1).

## 4 Discussion

This pediatric single-center retrospective cohort study describes our institutional experience with anticoagulation for patients with ICT, excluding those with an ICT in the setting of left ventricular assist device and extracorporeal membrane oxygenation. There was a wide variability in the duration of anticoagulation for ICT, with majority achieving PR or CR with 3-to-6 months of anticoagulation therapy and the median duration of anticoagulation was 138 days. Presence of a CVL was associated with a delayed time to ICT resolution. These findings may have significant implications in clinical decision making and underscore the need for larger studies to identify optimal duration of anticoagulation for CR of ICT.

In adults, the most common etiology for ICT is post myocardial infarction [11]. In pediatrics, the common etiologies are not well defined [12]. One pediatric case series showed that the risk of ICT was the highest in patients with dilated cardiomyopathy or post-Fontan operation [12]. Another study found a high incidence of ICT with the use of CVL; however, this cohort was limited to premature infants [13]. In our cohort, most ICT occurred in the setting of CVL, CHD, malignancy, and infections. This suggests that there may be a similar risk profile for ICT as seen in pediatric VTE [14,15].

In this cohort, presence of a CVL was the only significant factor associated with delayed resolution of the ICT. It is possible that the individuals with a CVL were overall more clinically complex, requiring longer hospital stays and all these factors may contribute to the slow resolution of the ICT. Additionally, given the retrospective nature of the study, we cannot differentiate if the underlying indication for a CVL was contributing to an overall refractory/enhanced prothrombotic state, or if the presence of CVL itself was the prothrombotic contributor to clot persistence. Larger prospective studies are required to further verify this observation. Poor LVEF has been recognized as a risk factor for left ventricular thrombosis with prevalence ranging from 2.1% to 7% in adults [16,17] and carries an increased risk of cardioembolic stroke [18]. We did not find the correlation of low LVEF and poor response to anticoagulation in our pediatric cohort. This could be explained by the number of the patients who had a decreased LVEF were also patients who had died (*n* = 7/17, 41.2%), and therefore, a resolution could not be observed. The 2022 American Heart Association guidelines recommend at least 3-to-6 months of therapeutic anticoagulation with discontinuation if LVEF improves to >35% or in the setting of major bleeding for adult patients [5], but pediatric clinical guidelines offer little discussion on anticoagulation under the setting of low LVEF. The advent of direct oral anticoagulants has made long-term anticoagulation more feasible. Hence, we recommend future prospective pediatric studies to focus on the utility of long-term anticoagulation for ICT associated with presence of a CVL and/or low LVEF and the impact of extended duration of therapy on patient and family quality of life. Analysis of other risk factors was likely limited by the small sample size of this study. ICT size has previously been implicated to correlate with duration of anticoagulation, with larger ICTs (>2 cm) requiring longer course than the traditional 3-to-6 months of anticoagulation [4,12,19]. However, we did not find this association in our cohort.

The most dreaded complication of ICT is thromboembolism, including stroke and PE [20,21]. In our cohort, the thromboembolic events noted were stroke. These adverse events have been most frequently described in people with left sided ICT, mobile thrombus, and/or large size of the thrombus [5,22]. We were unable to perform statistical analysis looking at the characteristics of ICT that were associated with thromboembolic complications due to small numbers in the cohort, but we observed that most of these events occurred in subjects with left sided mobile ICT. Future studies should stratify subjects based on the location of the intracardiac thrombus to determine the optimal duration of anticoagulation to prevent thromboembolic adverse events.

Our study has inherent limitations due to the retrospective nature of the study which makes it difficult to directly associate clinical risks (co-morbid conditions) and therapies with the outcomes. We relied on echocardiogram reports to determine the presence of ICT and may have failed to capture ICTs that were identified on cardiac MRI or other imaging. Owing to the small sample size of our cohort, we were limited in our ability to assess additional covariates or analyze multiple risk factors using multivariable regression analysis. Additionally, since there is no standardized protocol regarding when to repeat echocardiogram for repeat assessment of ICT, the decision was as per the treating physician’s discretion, and this may have resulted in variability in assessment of response. We did not collect timing of all the echocardiograms, and data collection was limited to the echocardiograms with findings of PR or CR, and the last echocardiogram during study period. Lastly, follow-up period was variable for the patients; hence it is possible that some of these patients were censored prior to seeing the optimal response to anticoagulation. We acknowledge that this is a single-center study that may affect the generalizability of these findings; hence, a multicenter study should be pursued to validate and build upon these observations to determine the optimal duration of anticoagulation for ICT.

In summary, we describe a cohort of children and young adults with ICT and demonstrate a wide variability in the duration of anticoagulation for ICT. Majority of the patients respond to 3-to-6 months of anticoagulation therapy. Our findings also suggest that those with a CVL may require a longer course of anticoagulation. We hope that these results will help clinicians make informed decisions regarding anticoagulation for ICT. Further larger scale studies are warranted to validate our findings and determine the optimal duration of anticoagulation, which can result in clinical practice change in this population.
